# Potential Interactions between the Autonomic Nervous System and Higher Level Functions in Neurological and Neuropsychiatric Conditions

**DOI:** 10.3389/fneur.2015.00182

**Published:** 2015-09-04

**Authors:** Andrea Bassi, Marco Bozzali

**Affiliations:** ^1^Clinical and Behavioural Neurology Laboratory, IRCCS Santa Lucia Foundation, Rome, Italy; ^2^Neuroimaging Laboratory, IRCCS Santa Lucia Foundation, Rome, Italy

**Keywords:** autonomic nervous system, cognitive, neurological disorders, orthostatic hypotension, heart rate variability, baroreflex sensitivity

## Abstract

The autonomic nervous system (ANS) maintains the internal homeostasis by continuously interacting with other brain structures. Its failure is commonly observed in many neurological and neuropsychiatric disorders, including neurodegenerative and vascular brain diseases, spinal cord injury, and peripheral neuropathies. Despite the different underlying pathophysiological mechanisms, ANS failure associates with various forms of higher level dysfunctions, and may also negatively impact on patients’ clinical outcome. In this review, we will discuss potential relationships between ANS and higher level dysfunctions in a selection of neurological and neuropsychiatric disorders. In particular, we will focus on the effect of a documented fall in blood pressure fulfilling the criteria for orthostatic hypotension and/or autonomic-reflex impairment on cognitive performances. Some evidence supports the hypothesis that cardiovascular autonomic failure may play a negative prognostic role in most neurological disorders. Despite a clear causal relationship between ANS involvement and higher level dysfunctions that is still controversial, this might have implications for neuro-rehabilitation strategies aimed at improving patients’ clinical outcome.

## Introduction

The autonomic nervous system (ANS) acts as an inside control system, which functions largely below the level of consciousness to regulate and coordinate bodily homeostatic functions and visceral adjustment under physical and mental stress. ANS outputs are based on secretory activity of glands and contraction of smooth muscles, while inputs mainly derive from afferent sensations arising from visceral receptors. In its peripheral section, the ANS can be functionally divided into parasympathetic and ortho-sympathetic branches, with additional components such as the enteric system (1). In the brainstem, there are located the principal ANS structures for the control of cardio-respiratory functions, which mediate vasomotor activity and specific reflexes, such as coughing, sneezing, vomiting, and swallowing (2). Just above the brainstem, within the diencephalon, the hypothalamus acts as an integrator for several autonomic functions, by linking together the central nervous system (CNS) and the endocrine system through the pituitary gland. It also receives inputs from the limbic system thus supporting a number of higher level functions, including memory, emotion processing, behavior, and motivation (3). Within the CNS, the so-called central autonomic network (CAN) has been identified as the top level system of regulation and includes the insular cortex and amygdala, the hypothalamus, the periaqueductal gray matter, the parabrachial complex, the nucleus of the tractus solitarius, and ventrolateral medulla (4, 5). These same brain regions are well known to be also implicated in cognitive functions, such as conflict monitoring, response inhibition, and interference resolution (6). Despite still being controversial, cardiac autonomic dysregulation (CAD) and cognitive decline have been reported in association with various conditions, such as neurodegenerative disorders with or without autonomic failure (AF) (7–9). Recent studies have also shown that brain and spinal cord injuries (mainly due to ischemic stroke) are frequently associated with cardiac autonomic unbalance, and that such an association may negatively affect patients’ rehabilitation outcome (10). It has been hypothesized that a major role in this association is played by cognitive impairment (CI), which may be partially due to cardiovascular dysregulation (11). As detailed below, there are several ways to explore the efficiency of the ANS *in vivo*. With these concepts in mind, this review aims at exploring the pathophysiological implication of ANS in higher level dysfunctions occurring in patients with neurological and neuropsychiatric diseases. Although this topic is still unexplored and controversial, this paper attempts to highlight the potential relationships between ANS and higher level dysfunctions in a selection of neurological and neuropsychiatric disorders.

## Autonomic Failure and Cardiovascular Autonomic Dysregulation

Symptoms referred to AF can be transiently observed in normal subjects and may be regarded as para-physiological individual features. On the other hand, recurrent or permanent symptoms of AF are commonly observed in various acute and chronic conditions, including neurological and non-neurological disorders. AF can therefore be considered as a pathophysiological substrate common to different clinical conditions and its acute/chronic characteristics and severity may differently impact on the patients’ clinical status. Orthostatic intolerance, change in sweating, gastrointestinal complaints, pupillary abnormalities, neurogenic bladder, and sexual dysfunctions or secreto-motor abnormalities are the most common features suggestive for AF (12, 13). Table 1 summarizes the main neurological and neuropsychiatric conditions in which autonomic symptoms may be observed. In some of them, such as in dementia with Lewy bodies (DLB), CI is a cardinal diagnostic feature (14). Nonetheless, dysautonomic symptoms are also frequently observed in DLB and may play a role in modulating patients’ CI. The link between the ANS and cognition lies on the evidence that patients suffering from neurogenic orthostatic hypotension (OH), in the presence of concomitant acute events (e.g., significant reduction of blood pressure), show a parallel worsening in cognitive performance (15). In this case, a bottom-up mechanism is responsible for cognitive dysfunctions. Previous neuroimaging studies in healthy subjects have indeed shown that individual differences in resting state or task-induced HRV correlate with brain activity in areas of the prefrontal cortex (16, 17) and with subjects’ cognitive performance (18). Consistently, other studies have shown a correlation between ANS efficiency and brain activity in regions traditionally devoted to various cognitive function (3, 19), but also implicated in mapping visceral responses. A direct association between ANS efficiency, cognitive performance, and regional brain activity has been recently demonstrated in healthy individuals, using task-related functional magnetic resonance imaging (fMRI) and parasympathetic stimulation of carotid baroreceptors (20). Similarly, Reyes del Paso et al. (1) demonstrated an effect of carotid baroreceptors’ stimulation in reducing pain perception.

**Table 1:** **Neurological and neuropsychiatric causes of autonomic failure**.

**1. Isolated autonomic failure**
1. Progressive
(a) Pure autonomic failure
2. Acute or subacute
(a) Autoimmune autonomic ganglionopathy
(b) Para-neoplastic autonomic neuropathy
**2. Progressive autonomic failure associated with parkinsonism, ataxia, or dementia**
1. Multiple system atrophy
2. Lewy body disorders
(a) Parkinson disease
(b) Dementia with Lewy bodies
3. Others
(a) Familial leukoencephalopathies
(b) Prion disorders
**3. Acute autonomic failure associated with acquired lesion of the central nervous system**
1. Acquired brain injury
2. Spinal cord injury
**4. Autonomic failure associated with peripheral neuropathy**
1. Chronic sensorimotor neuropathies
(a) Diabetes
(b) Amyloidosis
(c) Other metabolic disorders (vitamin B12 deficiency, uremia)
(d) Toxic neuropathies
2. Sensory ganglionopathies
(a) Sjögren’s syndrome
(b) Paraneoplastic
3. Distal painful neuropathies
(a) Diabetes
(b) Amyloidosis
(c) Idiopathic (sodium channelopathies)
(d) Infectious (Human immunodeficiency virus)
(e) Hereditary
(i) Hereditary sensory and autonomic neuropathy
(ii) Fabry disease
(iii) Sodium channelopathies
4. Acute or subacute motor polyradiculopathyorneuropathy
(a) Guillain–Barré syndrome
(b) Porphyria
5. Acute autonomic and sensory neuropathy
6. Ross syndrome (segmental anhidrosis, Adie pupils, and areflexia)

*Classification modified by Benarroch (13)*.

### Procedures to assess cardiac autonomic dysregulation

There are several methods to explore the top-down efficiency of the ANS *in vivo*. According to the 2011 Consensus Statement, the OH test is one of the most widely accepted. It is defined as a sustained reduction of systolic (at least 20 mmHg) or diastolic blood pressure (at least 10 mmHg) within 3 min after standing up or after a head-up tilt maneuver (at least 60°) (21). Alternative methods include the assessment of HRV and pressure regulation. Measures suggestive for CAD include the following: reduction in standard deviation normal to normal beat (SDNN) of HRV, unbalance of high/low frequency of HRV, decline of baroreflex sensitivity (BRS), and fault of nocturnal blood pressure regulation (22, 23).

The peripheral information is also known to induce autonomic changes within the CAN by Bottom/Up mechanism (24). However, there are no standardized measures available for this type of assessment.

### Autonomic failure and cognition

In healthy subjects, ANS reflex variability depends on gender and age. For instance, an attenuation of cardiovascular reflex is typically observed in young women and regarded, by some Authors, as a vagal-mediated cardio-protective phenomenon due to hormonal setting. Indeed, such a characteristic tends to disappear over aging (25–29). In turn, aging is also associated with a progressive decrease of the autonomic reflex, which is likely due to several factors, such as increased levels of oxidative stress, vascular stiffening, and decreased efficiency of cardiac cholinergic responsiveness (30). Aging associates with both cognitive modifications or impairments (31). In a selected sample of middle-aged subjects, a clear association was found between HRV and memory performance, which was independent from genetic and cardiovascular risk factors (32). Other studies indicate that vascular brain perfusion, which is also affected by sympathetic to parasympathetic balance, changes in the elderly. Saint Martin et al. (33) have investigated, in healthy populations, the potential relationship between vascular autonomic regulation and cognition, concluding that it is a risk factor for developing memory deficits in geriatric communities. Morphological changes in specific brain structures are also known to occur in the aging. Some of these structures, such as the brainstem, the insula, and the prefrontal cortex are implicated in the autonomic control, and again might contribute to physiological and pathological processes (34–36). In this complex picture, aging and pathology are clearly imbricated with each other. Based on a bottom-up mechanism, ANS dysregulation may contribute in determining successful or unsuccessful aging and in modulating the effect of diseases which affect cognition (37–39).

## Principal Neurological Causes of AF and Cognition

Table 1 summarizes the current classification of the neurological and neuropsychiatric causes of AF. Here, we will briefly review the major clinical conditions by focusing on their relationship with CI.

### Isolated autonomic failure

Isolated autonomic failure (IAF) is mainly due to an autoimmune mechanism. It is typically characterized by the presence of AF without any remarkable involvement of the CNS, and may present with an acute or subacute/progressive course. In the latter case, when affecting elderly individuals, IAF needs to be distinguished from the most common neurodegenerative diseases.

In patients with IAF, data on cognitive functions have been recently published by Guaraldi et al. (40). Transient worsening in executive functions was observed concomitantly with a fall in blood pressure during head-up tilt; this new evidence suggests a bottom-up causality mechanism for this transient CI. Further studies are needed to clarify the long-term effects of this vascular dysregulation on cognition.

### Progressive autonomic failure associated with neurodegenerative diseases

Progressive autonomic failure associated with neurodegenerative diseases (PAaND) is a heterogeneous group of CNS disorders, all characterized by a progressive clinical course (41). From a pathological viewpoint, a group of these conditions are known as α-synucleinopathies, as they are all characterized by intra-nuclear deposition of α-synuclein, despite a different cellular and anatomical distribution of the damage. They include the multi-system atrophy (MSA) and the Lewy body disorders.

#### Multi-System Atropy

Multi-system atropy is a sporadic, progressive disorder with an incidence of 3/100,000 per year in over 50-year-old individuals (42). Clinically, MSA may be dominated by parkinsonism, cerebellar ataxia or pyramidal deficits (43). The anatomical distribution of the brain damage mainly involves the striatum, the substantia nigra, the pontine and inferior olivary nuclei, the cerebellum, and the premotor autonomic nuclei (44, 45). The presence of atrophy in the putamen, middle cerebellar peduncle and pons on MRI supports a diagnosis of possible MSA (46). T2-weigthed MRI hypointensity of the posterior putamen surrounded by a hyperintense lateral putaminal rim or the so-called “hot cross bun sign” are also characteristic for MSA (47). In MSA, an earlier and more severe occurrence of AF is known to be associated with a quicker disease progression and more severe disability (48–50). Brown et al. (51) has hypothesized that cardiovascular AF is an independent predictor for CI in patients with MSA and progressive supranuclear palsy.

#### Lewy Body Disorders

The Lewy body disorders are a continuum spectrum, ranging from Parkinson’s disease (PD) to DLB, whose different clinical expression is believed to be due to the anatomical distribution of the damage. Damage is not only present in the substantia nigra and in the association cortex, but involves also structures which are directly or indirectly connected with the ANS. Additionally, Lewy body disorders are always (i.e., DLB) or frequently associated to CI. From an autonomic perspective, the clinical expression of these disorders ranges from asymptomatic cases to those experiencing frequent syncopes caused by AF. Other conditions associated with a progressive AF include the adult-onset autosomal-dominant leukodystrophies and prion disorders. In a proportion of cases, the clinical features are similar to those observed in the α-synucleinopathies, including autonomic symptoms and CI (52–55). Structural brain imaging is an essential tool for the differential diagnosis between the different forms of PAaND. For instance, the DAT scan is an essential assessment in the suspicion of DLB, when extrapyramidal symptoms are not fully manifest. The association between CAD and CI in α-synucleinopathies is an issue of emerging interest with potentially relevant clinical implications. CI is indeed largely explained by cortical neurodegeneration. However, patients with similar levels of brain atrophy may differ from each other for the severity of cognitive decline and clinical evolution. Cardiac AF is also known to impact on patients’ cognition through mechanisms of vascular dysregulation.

In PD patients with cardiac AF, it has been described an increased risk for stroke and mortality (56–58). Moreover, a strict association has been reported between the severity of cardiac noradrenergic denervation and the occurrence of visual hallucinations and dementia in patients with PD (59). In this association between AF and disease severity in patients with Lewy body disorders, the CI (which is at least partially explained by AF) is likely to play a remarkable role. For instance, in PD, Kim and co-authors (7) have reported an association between measures of vascular dysregulation (i.e., OH, supine hypertension, and vascular white matter changes) and patients’ level of cognitive decline. The link between AF and cognition certainly involves the whole brain, but some areas, implicated in both ANS control and cognition may play a more specific role. A recent study found that OH specifically reduces the cerebral blood flow in the anterior cingulate gyrus, which is critical for the cognitive domains typically affected in Lewy body disorders (60, 61). A chronic disarrangement of cerebral blood flow regulation might therefore exacerbate or worsen patients’ cognitive decline (62, 63). This pathophysiological mechanism lies on evidence obtained in animal model research (64, 65). Another specific brain structure, targeted by a-synuclein pathology and involved in both, ANS control and higher level functions, is the reticular formation. It is known that a specific association exists between REM-behavioral disorders and DLB, for which cognitive fluctuations represent one of the cardinal diagnostic criteria (14) and CAD is also often present (66). So far, in familial leukoencephalopathies and prion diseases, a strict association between CAD and cognition needs to be demonstrated. Future studies focused on this issue are needed to address this point.

Overall, in PAaND, a clear bottom-up causality mechanism for CI cannot be delineated. Indeed, CI is part of CNS degeneration (either cortical or subcortical). Nevertheless, we speculate that CAD may modulate the cognitive effect of such neurodegeneration, as documented by transient worsening of patients’ performances. Again, the long-term contribution of ANS dysfunction on permanent impairments in cognition needs to be clarified.

### Acute autonomic failure associated with acquired lesions of the central nervous system

Acquired CNS injury causes neurological impairment with a clinical spectrum depending on lesion localization and extension. In this picture, the ANS may also be implicated. The most common etiologies of Acute Autonomic Failure Associated With Acquired Lesions of the Central Nervous System (AAaAL) are as follows: stroke, subarachnoid hemorrhage, anoxia, and trauma. When the clinical presentation includes CI (typically in acute conditions overlapped to chronic risk factors, such as hypertension), the neuropsychological profile is dominated by executive dysfunctions (9, 67). Despite still being unclear, the presence of CAD may significantly determine worsening in patients’ cognition (68). In support to this hypothesis, it has been shown that, in patients with acute brain injury, the presence of sympathetic hyperactivity associates with a poor clinical outcome (69). Unfortunately, there are only few studies that investigate the relationship between brain lesion, AF, and CI. This is mainly due to patients’ heterogeneity in terms of etiologies, anatomical distribution of damage, etc. A causal interpretation of CNS and ANS contribution to patients’ CI needs to be further investigated.

### Autonomic failure associated with peripheral neuropathy

The potential relationship between CI and AF as due to peripheral neuropathies has not been systematically investigated in the literature. This is probably due to the concomitant presence of CNS involvement in the most common peripheral neuropathies, such as the diabetes mellitus. In this case, CAD as well as CI may be due to vascular lesions which are commonly detected in the brain tissue of diabetic patients. These lesions, which are mainly located in the white matter tissue, may induce brain disconnection and secondary gray matter degeneration. On the other hand, peripheral neuropathies involve not only the sensory-motor but also the autonomic fibers, thus resulting in ANS dysregulation. ANS dysregulation may therefore take part in causing/modulating patients’ CI (54). Studies focusing on AF and CI in purely peripheral neuropathies (e.g., CIDP) might be helpful in clarifying this relationship.

## Discussion

In many neurological and neuropsychiatric conditions characterized by CI, AF may also be present. OH is the most common feature suggestive for ANS dysregulation, and should always be carefully investigated in all patients. For this assessment, as described above, there are various techniques to identify ANS dysfunction not only when it is symptomatic but also subclinical. Despite the fact that the exact relationship between CI and AF still remains to be fully clarified, it is reasonable to assume that AF may at least contribute in determining cognitive symptoms. This is somehow obvious for neurological conditions such as the PAaNDs, for which ANS dysfunction is an essential part of the clinical picture. On the other hand, this seems more controversial when considering other conditions. For instance, DLB is by definition dominated by CI, but cognitive fluctuations are also a cardinal symptom for the diagnosis of DLB (14). Fluctuations may be partially explained by AF which, in turn, may play a role in modulating patients’ CI. In other common conditions, such as cerebrovascular disorders, ANS implication remains substantially neglected. In these patients, different clinical outcomes may be observed, and ANS dysfunction may directly or indirectly play a role in modulating patients’ clinical prognosis. We believe that, similar to PAaND, the clinical prognosis might depend on the presence of CI as caused by clinical or subclinical ANS dysfunction. There is also some emerging evidence that ANS dysregulation may be implicated in the unsuccessful aging and other degenerative forms of cognitive decline with no obvious autonomic impairment, such as Alzheimer’s disease. In these cases, subliminal symptoms should be explored by using measures which are more sensitive than the head-up tilt test, namely BRS and HRV (70–72). Meel-van den Abeelen et al. (15) have reported a direct association between BRS and cognitive performance in healthy elderly subjects as well as in patients with Alzheimer’s disease at different clinical stages (73–75).

In conclusion, the autonomic impairment especially in subclinical states is present in several pleiotropic neurological perturbations associated with CI. The most likely scenario is that there is a reciprocal relationship between the status of the ANS and central cognitive functionality. Considering the contribution of ANS dysfunctions will open new perspective for pharmacological and non-pharmacological interventions in several neurological and neuropsychiatric disorders.

## Conclusion

The implication of ANS in cognition seems to be a critical aspect in more and more neurological conditions. The autonomic impairment, at state of current knowledge, is associated in neuropsychiatric disorders with CI without a direct causal relationship. Despite the absence of conclusive data, this relationship deserves attention from both researchers and clinicians. Although it is still largely unexplored, this is indeed an interesting field not only for speculative reasons but also for potentially improving patients’ prognosis and for setting up more appropriate programs of neuro-rehabilitation. In order to fully clarify the relationship between CAD and CI, longitudinal studies are needed based on the use of standardized procedures for clinical and subclinical assessment of ANS dysregulation.

## Conflict of Interest Statement

The authors declare that the research was conducted in the absence of any commercial or financial relationships that could be construed as a potential conflict of interest.
